# ET-1 Plasma Levels, Aqueous Flare, and Choroidal Thickness in Patients with Retinitis Pigmentosa

**DOI:** 10.1155/2015/292615

**Published:** 2015-06-02

**Authors:** Ernesto Strobbe, Mauro Cellini, Michela Fresina, Emilio C. Campos

**Affiliations:** Ophthalmology Unit, Department of Experimental, Diagnostic, and Specialty Medicine, Alma Mater Studiorum, University of Bologna, Via Palagi 9, 40138 Bologna, Italy

## Abstract

*Purpose*. To assess endothelin-1 (ET-1) plasma levels, choroidal thickness, and aqueous flare in patients with early stage retinitis pigmentosa (RP) and to search for possible correlations. *Methods*. We compared 24 RP patients with 24 healthy controls. Choroidal thickness and aqueous flare were measured, respectively, by using a spectral domain optical coherence tomography and a laser flare-cell meter, whereas plasma samples were obtained from each patient to evaluate ET-1 plasma levels. *Results*. Notably, RP subjects showed significantly increased ET-1 plasma levels and reduced choroidal thickness compared with controls: 2.143 ± 0.258 versus 1.219 ± 0.236 pg/mL, *P* < 0.002, and 226.75 ± 76.37 versus 303.9 ± 39.87 *μ*m, *P* < 0.03, respectively. Higher aqueous flare values were also demonstrated in RP compared to controls: in detail, 10.51 ± 3.97 versus 5.66 ± 1.29 photon counts/ms, *P* < 0.0001. Spearman's correlation test highlighted that the increase of ET-1 plasma levels was related with the decrease of choroidal thickness (*r* = −0.702; *P* < 0.023) and the increase of aqueous flare (*r* = 0.580; *P* < 0.007). *Conclusions*. Early stage RP patients show a breakdown of blood-ocular barrier and increased ET-1 plasma levels and these findings may contribute to the reduction of choroidal thickness.

## 1. Introduction

Retinitis pigmentosa (RP) is a genetically heterogeneous hereditary disorder characterized by night blindness and progressive concentric visual field restriction, which may lead to severe central vision impairment due to degeneration and loss of photoreceptors and retinal pigment epithelium [1].

On the other hand, haemodynamic studies have demonstrated that RP patients show ocular blood flow disturbances, not only in retina and choroid but also in the retrobulbar vessels [2–4], and this finding may potentially contribute to the retinal damage. Moreover, increased endothelin-1 (ET-1) plasma levels have been described repeatedly in RP [5–7], and this increase might play a key role in determining vasoconstriction and ischemia, reducing ocular blood flow [8, 9], and leading to worsening of the abiotrophic process, as previously reported by our group in early stage RP [10].

Finally, alterations of the blood-ocular barrier and signs of intraocular inflammation, including cystoid macular edema, have been demonstrated in RP patients, by clinical examination and by fluorophotometric studies [11–13], and notably, Küchle and associates showed that RP eyes have increased aqueous flare values compared with controls that is strictly associated with cystoid macular edema [14].

In an attempt to establish better whether a relationship between ocular inflammation and haemodynamic alterations may exist and whether RP subjects may show some abnormalities in the choroid, we investigated the status of the blood-ocular barrier in early stage RP subjects by using the laser flare-cell meter and evaluated subfoveal choroidal thickness (SCT) by using spectral domain optical coherence tomography (SD-OCT).

## 2. Materials and Methods

The current study was an observational, case-control, single-center study performed in accordance with the tenets of the Declaration of Helsinki, was conducted between October 2012 and June 2014 at the S. Orsola-Malpighi Hospital in Bologna, and was reviewed and approved by the Institutional and Ethical Committee of University of Bologna.

Each patient signed an informed consent form, before being enrolled in the study, after a full explanation of the aim of the study and of the procedures.

24 patients aged 25 and 42 years, affected by early stage RP, were enrolled from a cohort of 58 RP subjects followed up by our center and compared with 24 age- and sex-matched healthy controls, aged between 28 and 45 years.

The diagnostic criteria for the early stage of RP we followed up are those described by Hamel in 2006 [15]: night blindness, peripheral visual field defects, normal or subnormal visual acuity, color vision and life habits, modest attenuation of retinal arterioles, normal or fairly pale optic disc, absent or rare peripheral bone spicule-shaped pigment deposits, and a decrease in maximum electroretinogram (ERG) amplitude. Thus, we selected only young RP patients with preserved visual function, nyctalopia, peripheral visual field defects, and decreased but not extinct ERG and excluded those who had systemic diseases, such as systemic hypertension, diabetes, and cardiovascular disease, were taking any medications, had cystoid macular edema demonstrated with SD-OCT and advanced posterior subcapsular cataract, or were syndromic RP patients.

All participants underwent a complete ophthalmological evaluation, including visual acuity, Goldmann applanation tonometry, and slit-lamp examination of anterior and posterior segment. A visual field test, an ERG, and assessment of choroidal thickness, aqueous flare, and ET-1 plasma levels were also performed.

### 2.1. Visual Field Test

Visual field test was performed by using standard automated perimetry with the Humphrey 740 field analyzer 30.2 full threshold program (Humphrey Instruments Inc., San Leandro, CA, USA), and both mean defect (MD) and pattern standard deviation (PSD) were obtained using Humphrey STATPAC software and were expressed in decibels (dB).

### 2.2. Electroretinogram

Electroretinogram (ERG) was recorded by using RETIMAX Plus Advanced (CSO Ophthalmic, Florence, Italy). After pupil dilation with 1% tropicamide and topical anesthesia of the cornea with 0.4% oxybuprocaine hydrochloride, HK-Loop ERG electrodes, a ground electrode, and the reference electrodes were applied, respectively, to the ocular surface, to the forehead, and to the temporal region. Electrical impedance was smaller than 5 k*ω* for all electrodes. ERGs were recorded from both eyes simultaneously, according to International Society for Clinical Electrophysiology of Vision (ISCEV) standards [16], by using a standard flash strength of 2.5 cd·s·m^−2^. Band-pass filter was between 0.3 Hz and 300 Hz with an amplification of 5 k while artifactual signals were automatically removed. The amplitudes of b-wave and a-wave were measured from the baseline and were expressed in microvolts (*μ*V).

### 2.3. SD-OCT Image Acquisition and Analysis

SD-OCT is a noninvasive, objective technique that allows in vivo cross-sectional high resolution visualization of retina and choroid with a fast scanning speed [17]; however, to provide many details of the structure of the choroid, a new technique called enhanced depth imaging (EDI) has been developed in the recent years and described elsewhere [18, 19].

**Figure 1:**
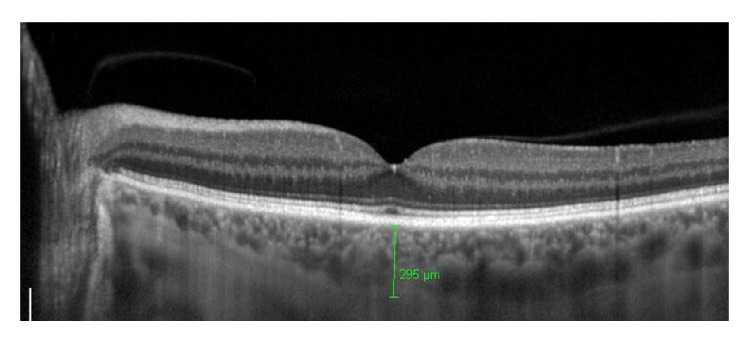
Representative enhanced depth imaging optical coherence tomography scan of a control subject. Choroidal thickness was measured as the distance between the hyperreflective line of the retinal pigment epithelium-Bruch's membrane complex and the innermost hyperreflective line of the choroid-sclera junction. For illustration purposes, the resultant images were reinverted.

We chose the horizontal scan running directly through the center of the fovea of both eyes of each patient by using SD-OCT (Heidelberg Engineering GmbH, Heidelberg, Germany) with the EDI technique and choroidal thickness was measured manually and was defined as the vertical distance from the hyperreflective line of the retinal pigment epithelium-Bruch's membrane complex to the innermost hyperreflective line of the choroidal scleral interface, exactly below the foveola. The values were expressed in micrometers (m*μ*) (Figure 1).

### 2.4. Endothelin-1 Determination

For ET-1 measurements the plasma samples were drawn from the antecubital vein and collected in a container with EDTA, cooled, and stored in ice. Subsequently, the samples were centrifuged at 4°C and frozen at −25°C. After centrifugation, the extraction was performed using a Sep-column containing C-18 (Peninsula Laboratories, Belmont, CA, USA) and ET-1 concentration was determined by using a commercial radioimmunoassay (RIA) kit (RIK-6901 Peninsula Laboratories, Belmont, CA, USA); after that samples and standards were firstly incubated with rabbit anti-ET-1 serum for 24 hours at 4°C. A second 24-hour incubation was performed after the addition of an iodinated tracer [^125^I]-ET-1 (Peninsula Laboratories, Belmont, CA, USA). Free and bound radioligands were separated with centrifugation and radioactivity in the precipitate was counted with an automatic gamma-counter (Packard Industries, Boonton, NJ, USA). ET-1 concentration was expressed in picogram/milliliter (pg/mL).

### 2.5. Aqueous Flare Measurement

Examination of the aqueous humor by slit-lamp biomicroscopy is the primary method by which ophthalmologists evaluate the severity of anterior segment inflammation, by counting the number of cells and estimating “flare,” that is, the amount of protein in the aqueous humor. Obviously, the ability to quantify inflammation relies on the experience of the examiner and this may result in substantial interobserver variability. For this reason, in the late 1980s, a laser flare-cell meter, an automated objective and noninvasive technique that enables rapid and reproducible measurement of cells and flare in the aqueous humor [20, 21], by providing information about the status of the blood-aqueous barrier (BAB), and is able to identify small changes in aqueous humor proteins during the course of a disease that are not apparent clinically by slit-lamp biomicroscopy, was developed.

The device we used to assess intraocular inflammation was the laser flare-cell meter FC-500 (Kowa Company, Ltd, Tokyo, Japan), which uses a diode laser that is projected into the anterior chamber to scan a measurement window of 0.3 × 0.5 mm over 0.5 seconds [20], and the amount of backscattered light by protein particles in the aqueous humor, which is proportional to the concentration and size of proteins, is detected by a photomultiplier and processed by a computer. The average of signals coming from above and below the measurement window (background signals) is subtracted from the signal obtained from inside the scanned window to provide a laser flare photometry measurement.

The accuracy and reproducibility of the method have been shown in several studies by different groups [20, 22, 23], the coefficient of variation is less than 10%, and measurements are independent of the examiner using the instrument. Seven measurements for each eye were obtained and averaged, and those with artifacts were eliminated. The results were expressed as photon counts per millisecond (pc/ms).

### 2.6. Statistical Analysis

All data were expressed as the mean ± standard deviation (SD) and only 1 eye, for each subject, was randomly selected for statistical analysis. The statistical analysis was performed with MedCalc 10.9.1 statistical program (MedCalc Software, Ostend, Belgium), to assess the differences between RP patients and controls by using the Wilcoxon rank-sum test. Spearman's correlation test was used to evaluate the relationship between choroidal thickness and aqueous flare and the association between ET-1 plasma levels and both choroidal thickness and subclinical ocular inflammation. *P* values less than 0.05 were regarded as being statistically significant.

## 3. Results

A total of 48 eyes from 24 RP patients (male-to-female ratio, 14 : 10; mean age, 33.8 ± 7.3 years) and 24 healthy subjects (male-to-female ratio, 12 : 12; mean age, 36 ± 6.8 years) were examined in this study (Table 1).

As regards RP patients, the limits of visual field test parameters we considered for inclusion in the study were MD > −5 dB and PSD > 2.5 dB, whereas the limits of ERG were b-wave amplitude < 50 *μ*V and a-wave amplitude < 35 *μ*V (in our lab, normal range for a-wave and b-wave was 56.87–72.02 and 35.37–40.78 *μ*V, resp.).

Clinically, no participants showed anterior chamber inflammatory reaction (cells and flare) or abnormalities in the lens and vitreous. Mild to modest attenuation of retinal arterioles, normal or fairly pale optic disc, and absent or rare peripheral bone spicule-shaped pigment deposits were also observed by slit-lamp biomicroscopic examination.

Age, best-corrected visual acuity, and intraocular pressure did not differ significantly between RP patients and healthy controls (*P* > 0.05), whereas there was a highly significant difference with regard to visual field parameters, MD (*P* < 0.006) and PSD (*P* < 0.001) and ERG; indeed, RP had peripheral visual field defects and decreased b-wave and a-wave amplitude compared to controls (*P* < 0.002 and *P* < 0.019, resp.). In addition, RP patients showed significantly higher ET-1 plasma levels and aqueous flare than controls, 2.143 ± 0.258 versus 1.219 ± 0.236 pg/mL (*P* < 0.002) and 10.51 ± 3.97 versus 5.66 ± 1.29 pc/ms (*P* < 0.0001), respectively, but also a significant reduction in choroidal thickness: 226.75 ± 76.37 versus 303.9 ± 39.87 *μ*m (*P* < 0.03) (Table 1).

**Figure 2:**
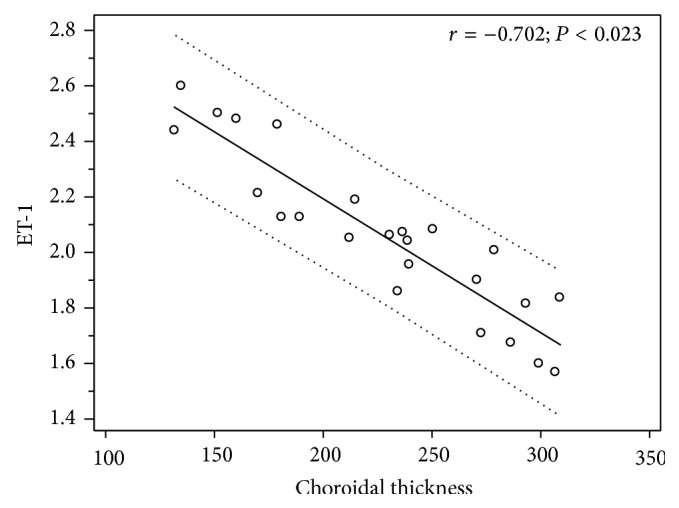
Scatterplot showing the correlation between ET-1 plasma levels (picogram/milliliter) and subfoveal choroidal thickness (micrometers) in patients with retinitis pigmentosa.

**Figure 3:**
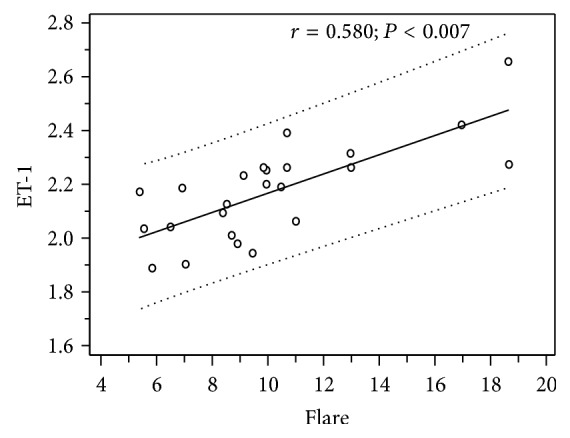
Scatterplot showing the correlation between ET-1 plasma levels (picogram/milliliter) and aqueous flare (photon counts/millisecond) in patients with retinitis pigmentosa.

Furthermore, Spearman's correlation test highlighted that the increase of ET-1 plasma levels in RP was related with the decrease of choroidal thickness (*r* = −0.702; *P* < 0.023; Figure 2) and the increase of intraocular inflammation, represented by aqueous flare (*r* = 0.580; *P* < 0.007; Figure 3), whereas no statistically significant correlation between aqueous flare and choroidal thickness (*r* = −0.308; *P* = 0.124) was reported.

## 4. Discussion

Retinitis pigmentosa is a group of inherited disorders characterized by progressive peripheral visual field loss, abnormal ERG responses and variable clinical presentation, severity, age of onset, and progression and may lead to central vision loss because it diffusely involves photoreceptors and retinal pigment epithelium (RPE) [1].

To the best of our knowledge, no data have been published concerning the relationship between intraocular inflammation and ET-1 plasma levels in RP patients.

Our results demonstrate that subjects affected by early stage RP with preserved central visual acuity have an 86% increase in aqueous flare values, a 34% decrease in choroidal thickness, and statistically significant higher ET-1 plasmatic levels compared with healthy controls.

The increase in aqueous flare reflects a disruption of the BAB, which allows leakage of serum proteins, as well as inflammatory molecules and cells, into the anterior segment, by causing a change in aqueous protein composition and concentration. By means of the noninvasive laser flare-cell meter that may provide an objective assessment of the status of the BAB [20], we showed that RP leads to a breakdown of the BAB that causes a local anterior subclinical inflammation that is not apparent clinically by slit-lamp biomicroscopy. This finding is in agreement with previous studies; indeed, fluorophotometric studies reported increased amount of fluorescein leakage into the vitreous of eyes with RP [12], whereas Küchle and associates [14] demonstrated that subjects affected by RP have higher aqueous flare values compared with healthy controls. Finally, Yoshida and coworkers [24] showed that aqueous flare is increased in RP patients and negatively correlates with visual function in phakic eyes.

The exact mechanism by which ocular inflammation occurs in RP patients is not clear, but two reasons may be postulated: firstly, most dystrophic and degenerative diseases are accompanied by low-grade inflammation; it is well known that increased retinal lipofuscin fluorophores in RP may determine damage, disturbed polarity, death of RPE, and apoptosis of photoreceptors [25].

In response to this stimulation, RPE synthesizes and releases a large variety of inflammatory molecules such as cytokines and chemokines [26], which, in turn, promote the recruitment of inflammatory cells that leak into the vitreous and may reach the aqueous, as there is no barrier separating the posterior from the anterior segment [27, 28], with a resulting enhanced aqueous flare. Secondly, as blood retinal barrier breakdown occurs both in retinal vessels and in RPE [29, 30], even BAB may be affected, leading to increased amount of proteins into the aqueous.

The thinnest choroidal thickness values we measured in RP patients compared with controls (*P* = 0.03), by using EDI SD-OCT, are in agreement with those already demonstrated by Dhoot et al. [31] with the same objective technique. It is possible that RPE and photoreceptor degeneration may result in choroidal thinning due to choriocapillaris atrophy [32, 33]; in animal models, it has been demonstrated that these cells are necessary for choroidal maintenance and thickness by producing several factors, such as vascular endothelial growth factor [34].

However, we reported choroidal thinning in patients with preserved visual acuity, suggesting that, probably, abnormalities in choroidal circulation and flow are the primum movens of photoreceptor degeneration. Indeed, both retinal and choroidal blood flow have previously been shown to be reduced in patients with RP, by using color Doppler imaging and laser Doppler flowmetry [2–4, 35, 36], and fluorescein angiography studies further support that choroidal circulation and volume are reduced and disturbed in RP [37–39].

Finally, these patients showed increased ET-1 plasma levels, and this finding was well related with the decrease of choroidal thickness and the increase of intraocular subclinical inflammation. Such an increase in ET-1 plasma levels in RP patients has been described in previous studies [5–7], but not confirmed by all researchers [40]. Different disease heterogeneity, stages of RP, race, sample size, and the method used for the ET-1 determination may justify contrasting results and different values reported in published data [7].

Currently, the exact mechanism why ET-1 levels are increased is not completely elucidated, but possible explanations may be formulated. Whether ET-1 is mainly produced by vascular endothelial cells, also several kinds of cells may synthetize and secrete ET-1 when they are under stress conditions, such as hypoxia and oxidative stress, and interestingly, it has been widely suggested that oxidative stress is a typical finding in RP and possibly contributes to its pathogenesis [41].

On the other hand, some authors have hypothesised a primary vascular dysregulation syndrome [42, 43] that might be the cause for the observed findings in blood flow reduction and increase in ET-1 and could explain all the signs and symptoms both in eye and in the body of RP patients. Indeed, Cellini and coworkers demonstrated, by using laser Doppler flowmetry, that blood flow abnormality in RP patients was not confined to the eye but also occurred in the peripheric circulatory system [5]. Thus, the observed increase of ET-1 plasma level might be secondary to vascular dysfunction and/or subclinical systemic inflammation.

Notably, we reported a negative association between choroidal thickness and ET-1 (*r* = −0.702; *P* < 0.023) and hypothesize that the reduction of choroidal thickness may be determined by the vasoconstrictive effect of ET-1, which may lead to vasospasms and unstable ocular blood supply, by altering the regulation of choroidal perfusion with a resulting relative ischemia.

There was also a significant positive correlation between aqueous flare and ET-1 plasmatic levels (*P* < 0.007) in RP patients. It is interesting to notice that RP subjects have an imbalance of the antioxidant-oxidant status in the peripheral blood [41] that leads to an increase in free radicals, chronic oxidative stress, and subclinical inflammation which may stimulate cells to secrete more ET-1, as in a vicious circle; on the other hand, increased ET-1 may, in turn, lead to vascular dysregulation and hypoxic stress with resulting activation of inflammatory pathways.

Independent of the cause of the ET-1 increase in the plasma, this increase has consequences on ocular blood impairment, and the thinned choroid reported in our patients may be a manifestation of this decreased flow.

In addition, choroidal thickness did not correlate with the extent of subclinical inflammation of the anterior chamber; this means that BAB breakdown does not directly influence the thinning of the choroid.

This study has some limitations. Firstly, the sample size was small because participants were accurately selected among young early stage RP patients, to limit confounding factors, such as age, systemic diseases, and different stages of RP, which could affect plasmatic ET-1 levels. Secondly, we did not evaluate intraocular ET-1 concentration for obvious ethical reasons. Thirdly, the retrospective nature of this study represents another limitation. Finally, we have no data on the systemic inflammatory status of the affected patients to strengthen our results. Larger evaluation of RP patients to investigate the correlation between vascular dysregulation and inflammation will help shed further light on the key role of these elements in RP.

## 5. Conclusions

To our knowledge, this is the first study that demonstrates a correlation between ocular inflammation and ET-1 plasma levels in early stage RP patients. This means that increased ET-1 plasma levels and subclinical inflammation may interact to play a key role in the impairment of choroidal blood flow, supply, and thickness which lead to an increase in free radicals and chronic oxidative stress.

Although our results require further confirmation and investigation, this study might provide reasonable data about the possibility to use antagonists of ET-1 and anti-inflammatory molecules together with antioxidants with the purpose of improving the ocular blood flow to ameliorate microvascular function and reduce the progression of the disease.

## Figures and Tables

**Table 1:** Demographic, ocular parameters, and ET-1 plasma levels in patients with retinitis pigmentosa and healthy controls.

	Retinitis pigmentosa	Controls	*P* < 0.05
Age, years	33.8 ± 7.3	36.0 ± 6.8	0.155
Visual acuity, decimals	0.95 ± 0.07	0.97 ± 0.04	0.235
IOP, mmHg	15.8 ± 2.5	15.6 ± 2.3	0.81
SAP-PSD, dB	6.09 ± 4.22	1.98 ± 0.98	**0.001**
SAP-MD, dB	−7.90 ± 1.75	−1.95 ± 0.83	**0.006**
ERG b-wave, *μ*V	45.08 ± 8.24	65.36 ± 9.84	**0.002**
ERG a-wave, *μ*V	28.13 ± 5.77	38.16 ± 5.57	**0.019**
ET-1, pg/mL	2.143 ± 0.258	1.219 ± 0.236	**0.002**
Choroidal thickness, *μ*m	226.75 ± 76.37	303.9 ± 39.87	**0.023**
Aqueous flare, pc/ms	10.51 ± 3.97	5.66 ± 1.29	**0.0001**

Note: values are presented as means ± SD; *n* = 24 per group.

IOP = intraocular pressure; SAP = standard automated perimetry; PSD = pattern standard deviation; MD = mean defect; dB = decibel; ERG = electroretinogram; *μ*V = microvolt; ET-1 = endothelin-1; pg/mL = picogram/milliliter; *μ*m = micrometer; pc/ms = photon counts/millisecond.
